# SJNNV down-regulates RGNNV replication in European sea bass by the induction of the type I interferon system

**DOI:** 10.1186/s13567-015-0304-y

**Published:** 2016-01-08

**Authors:** Carlos Carballo, Esther Garcia-Rosado, Juan J. Borrego, M. Carmen Alonso

**Affiliations:** Departamento de Microbiología, Facultad de Ciencias, Universidad de Málaga, 29071 Málaga, Spain; IFAPA centro El Toruño, Junta de Andalucía, El Puerto de Santa María, Cádiz Spain

## Abstract

European sea bass is highly susceptible to the betanodavirus RGNNV genotype, although the SJNNV genotype has also been detected in this fish species. The coexistence of both genotypes may affect the replication of both viruses by viral interaction or by stimulation of the host antiviral defense system in which the IFN I system plays a key role. IFN I triggers the transcription of interferon-stimulated genes, including Mx genes, whose expression has been used as a reporter of IFN I activity. The present study evaluated the effect of a primary exposure to an SJNNV isolate on a subsequent RGNNV infection and analyzed the role of the IFN I system in controlling VNNV infections in sea bass using different in vivo approaches. VNNV infection and Mx transcription were comparatively evaluated after single infections, superinfection (SJ+RG) and co-infection (poly I:C+RG). The single RGNNV infection resulted in a 24% survival rate, whereas the previous SJNNV or poly I:C inoculation increased the survival rate up to 96 and 100%, respectively. RGNNV replication in superinfection was reduced compared with RGNNV replication after a single inoculation. Mx transcription analysis shows differential induction of the IFN I system by both isolates. SJNNV was a potent Mx inducer, whereas RGNNV induced lower Mx transcription and did not interfere with the IFN I system triggered by SJNNV or poly I:C. This study demonstrates that an antiviral state exists after SJNNV and poly I:C injection, suggesting that the IFN I system plays an important role against VNNV infections in sea bass.

## Introduction

Viral nervous necrosis (VNN) is a disease that affects a wide range of marine and freshwater fish species, including the European sea bass (*Dicenthrarchus labrax*). This fish species is especially susceptible to this viral disease at larval and juvenile stages, although mortalities in adult specimens have also been recorded [1]. Affected animals display various neurological symptoms such as abnormal swimming behavior, anorexia, or altered floatability.

VNN is caused by the viral nervous necrosis virus (VNNV), which belongs to the *Betanodavirus* genus, *Nodaviridae* family. The viral genome is composed of 2 single-stranded, positive-sense RNA molecules [2]. RNA1 (3.1 kb) encodes the RNA-dependent RNA-polymerase (RdRp), and RNA2 (1.4 kb) encodes the capsid protein (CP). In addition, VNNV has a subgenomic transcript of the RNA1 segment, named RNA3, which contains an open reading frame (ORF) that encodes two non-structural proteins.

Betanodaviruses have been classified into the following four genotypes based on the sequence of the variable T4 region within the RNA2 segment [3]: striped jack nervous necrosis virus (SJNNV), red-spotted grouper nervous necrosis virus (RGNNV), tiger puffer nervous necrosis virus (TPNNV) and barfin flounder nervous necrosis virus (BFNNV).

Although the SJNNV and RGNNV genotypes have been detected in European sea bass [4–8], RGNNV appears to be the only genotype that causes high mortalities in this fish species [8], suggesting that both genotypes may interact differentially with the sea bass antiviral immune system. In addition, both genotypes may coexist in the same specimen [9], which may lead to the reassortment of both viral segments. Indeed, reassortant betanodaviruses have been isolated from sea bass, sea bream (*Sparus aurata*) and Senegalese sole (*Solea senegalensis*) [7, 10], suggesting that dual infections may be frequent events. In addition, viral coexistence may affect the multiplication of the viruses involved, which may be caused by the interaction of both viruses during their replication cycle or by the induction of antiviral factors such as the factors involved in the type I interferon system (IFN I) [11, 12].

IFN I triggers an antiviral state by stimulating the expression of interferon-stimulated genes (ISGs), which include the genes that encode the Mx proteins, with proven antiviral activity in multiple fish species [13, 14]. Mx proteins belong to the dynamin superfamily of high molecular weight GTPases, which are involved in intracellular membrane remodeling and intracellular trafficking [15]. Mx gene transcription has been used in the present study as an IFN I system stimulation reporter. IFN I synthesis can be induced either by viral infection or by polyinosinic-polycytidylic acid (poly I:C), a synthetic double-stranded RNA molecule. Indeed, previous studies have demonstrated an antiviral state in several fish species after poly I:C treatment [16, 17].

Several in vivo studies have examined the coexistence of different viruses in a single fish [11, 12, 18–20]; however, to our knowledge, the only report studying the coinfection of two types of betanodaviruses has been performed using cell culture [21]. The current work constitutes a step forward in the study of nodavirus coexistence. The aim of the present work was to determine the effect of SJNNV exposure on a subsequent RGNNV infection and to evaluate the role of the IFN I system in modulating the RGNNV infection in experimentally challenged European sea bass.

## Materials and methods

### Virus and cell culture

The following VNNV isolates were used in this study: (1) ERV378/102-5/04 (RGNNV genotype, isolated from sea bass) and (2) SJ93Nag (SJNNV genotype, reference strain). Both viruses were propagated using the E-11 cell line [22]. The E-11 cells were grown at 25 °C in Leibovitz (L15) medium (Gibco, Carlsbad, CA, USA) supplemented with 10% fetal bovine serum (FBS, Gibco) and 1% penicillin–streptomycin (Gibco). After virus inoculation, the cells were maintained at 25 °C in L15 medium containing 2% FBS and 1% penicillin–streptomycin until the cells displayed extensive cytopathic effects (CPE). Viral titration was performed in 96-well plates (Nunc Thermo Scientific, Lanenselbold, Germany) using the 50% tissue culture infectious dose (TCID_50_) method [23].

### Fish infections

Juvenile European sea bass were acclimatized at the aquaculture facility of the University of Malaga (Spain). The animals were fed once a day and maintained in 800-L tanks with continuous aeration and a closed water system. Temperature (25 °C) and salinity (37 g/L) were maintained during all the experiments.

Prior to the challenges, 9 fish were randomly collected from each tank, and their brains and eyes were analyzed according to Lopez-Jimena et al. [9] to discard a possible VNNV asymptomatic carrier state (data not shown).

### Challenge 1. Effect of SJNNV replication on a subsequent RGNNV infection

Juvenile European sea bass (weight between 10 and 15 g, *n* = 150) were intramuscularly (IM) injected with SJNNV 24 h before RGNNV inoculation (SJ+RG group). In addition, the three following control groups were considered (*n* = 150 per group): (1) L15+L15 (negative control: first and second injection with L15 medium); (2) L15+RG (control of the normal course of the RGNNV infection: first inoculation with L15, second inoculation with RGNNV) and (3) SJ+L15 (control of the normal course of the SJNNV infection: first inoculation with SJNNV, second inoculation with L15). As in the experimental SJ+RG group, second inoculations were always 24 h after the first inoculation, and the viral dose used was 1 × 10^5^ TCID_50_/g.

Nine fish per group were randomly collected at different time points post-inoculation (pi) and were killed with an overdose of anesthetic (MS-222, Sigma, St. Louis, MO, USA). The fish used in this study were treated according to the Spanish directive (RD 53/2013, BOE no. 34) [24]. Nervous system organs (pooled eyes and brain) and head kidneys from three animals were aseptically collected and pooled separately. Thus, a total of three samples comprising tissues from three animals were obtained per organ, group and time pi. These samples were immediately frozen in liquid nitrogen and stored at −80 °C until use. Samples for virological analyses (pooled brain and eyes) were collected at 12 h, 3, 7 and 29 days after the second injection. Samplings for Mx transcription analyses (head kidney) were performed at 3, 12 and 24 h after viral inoculation in the single-inoculated groups, and at the same time points after the second inoculation in the groups that were inoculated twice. The methodology used to quantify the viral genome and Mx mRNA is described below.

A group of 50 fish per treatment were maintained for 30 days to estimate the accumulated survival rate according to the Kaplan–Meier estimator [25]. Mortality was recorded daily, and dead fish were removed and stored at −80 °C for virological analysis. The presence of the virus in dead fish was confirmed by inoculating homogenates of nervous tissue (pooled brain and eyes) onto E-11 cell monolayers. Virus titration from these homogenates was performed via the TCID_50_ method.

### Challenge 2. Influence of a previous poly I:C treatment on RGNNV infection

A previous challenge was conducted to determine the time of maximum Mx transcription after poly I:C stimulation. Thus, the animals (30 g, average weight) were IM-inoculated with poly I:C (Sigma, 15 mg/kg), and individual head kidneys were collected and processed to conduct the Mx transcription analyses as described below. The samples were collected at 0, 4, 8, 10, 12 and 24 h post-injection (hpi).

Regarding challenge 2, juvenile sea bass (30 g, average weight, *n* = 45 per group) were injected with poly I:C 12 h before they were infected with the RGNNV isolate (poly I:C+RG group). In addition, a control group, in which poly I:C was replaced by L15, was also considered (L15+RG group). All the inoculations were performed by IM injection using a viral dose of 1 × 10^5^ TCID_50_/g and/or 15 mg/kg poly I:C.

Head kidneys from 6 animals were aseptically collected at 12 h after the first inoculation to assess Mx transcription induced by poly I:C at the time of the second inoculation. The remaining animals were maintained for 30 days to obtain the accumulated survival rate.

### Challenge 3. Interaction between the IFN I system and the RGNNV infection

This study was conducted with juvenile European sea bass specimens (10 g, average weight, *n* = 30) injected consecutively with poly I:C and RGNNV. In addition, the following control groups were analyzed: (1) poly I:C+L15 (poly I:C-stimulated animals) and (2) L15+RG (RGNNV-infected animals). The fish were IM-injected with a viral dose of 1 × 10^5^ TCID_50_/g and/or 15 mg/kg poly I:C. Mx transcription was quantitatively analyzed in head kidneys sampled at 12, 24 and 48 hpi (three individual samples per sampling time), as described below.

### Sample processing

Pooled nervous organs were homogenized in L15 medium (20%, w/v) containing 1% penicillin–streptomycin and 2% FBS. Homogenates were centrifuged twice at 7500 ×* g* at 4 °C for 15 min. In total, 200 µL of each homogenate was used for total RNA extraction with TRIzol (Invitrogen, Life Technologies, Carlsbad, CA, USA) following the manufacturer’s instructions. The remaining volume of each homogenate was treated with 100 µL/mL penicillin–streptomycin at 4 °C overnight, centrifuged twice at 7500 ×* g* at 4 °C for 15 min, and was used for virus titration with E-11 cells as described below.

Head kidney samples were homogenized in 1 mL of TRIzol for total RNA extraction as described above. Genomic DNA was degraded by treating the total RNA with RNase-free DNase I (Roche, Basel, Switzerland) following the manufacturer’s instructions.

The total RNA concentration was determined at 260 nm using the ND-1000 system (NanoDrop Thermo Scientific, Wilmington, USA). The RNA was stored at −80 °C until use, and the cDNA synthesis was performed using the Transcriptor First Stand cDNA Synthesis Kit (Roche) with random hexamers and 1 µg of total RNA. The cDNA concentration was determined at 260 nm using the ND-1000 system, and the cDNA was stored at −20 °C until further use.

### Viral quantification

The viral titer was calculated via the TCID_50_ method on semiconfluent monolayers of E-11 cells seeded onto 96-well plates (Nunc Thermo Scientific) according to Lopez-Jimena et al. [21]. Viral titers were expressed as TCID_50_/g of nervous tissue.

The independent titer of each genotype in the superinfected group within challenge 1 (SJ+RG) was calculated after viral neutralization using the following polyclonal antibodies: (1) anti-NNV ab26812 (Abcam), which neutralizes the RGNNV genotype, and (2) an anti-SJNNV antibody developed in the rabbit (kindly provided by Dr. T Nakai, University of Hiroshima, Japan) for SJNNV neutralization.

The homogenates were mixed (1:1, v/v) with a 1:100 dilution of the antibody (in L15 supplemented with 1% penicillin–streptomycin). The mixture was incubated for 1 h at 25 °C and subsequently inoculated onto semiconfluent E-11 cell monolayers seeded onto 96-well plates as previously described.

Viral genome quantification was performed following two absolute quantitative PCR (qPCR) protocols previously reported by Lopez-Jimena et al. [21, 26], which separately detected the RNA2 segment of the RGNNV and SJNNV genotypes.

Real-time PCR was performed with 75 ng of cDNA obtained from nervous tissue. The samples were analyzed in triplicate with the FastStart Universal SYBR Green Master (Rox) (Roche) in a final volume of 25 µL. The reactions were performed using the 7500 Real-time PCR System (Applied Biosystems) in 96-well plates as follows: one step at 50 °C for 2 min, one step at 95 °C for 10 min, followed by 40 cycles of 95 °C for 15 s and 60 °C for 1 min.

### Mx gene transcription

Mx mRNA was quantitatively analyzed by relative qPCR using the Mx-Fw/Rw primers reported by Chaves-Pozo et al. [27]. The 18S rRNA gene was used as an endogenous control [28].

Real-time PCR reactions (20 µL, final volume) were performed with 100 ng of head kidney cDNA, using the 7500 Real-time System (Applied Biosystems, CA, USA), and the FastStart Universal SYBR Green Master (Rox) in 96-well plates. The amplification profile was: 50 °C for 2 min, 95 °C for 10 min, followed by 40 cycles of 95 °C for 15 s, and 60 °C for 1 min. Relative Mx gene transcription was calculated by the 2^−ΔCt^ method [29].

### Statistical analyses

Mortality data were used to perform the accumulated survival curves with the IBM^*®*^SPSS^*®*^Statistics v21 software. The Breslow test was used to verify the equality of survivor functions between the experimental groups and challenges. Significant differences were considered at values of *p* < 0.05.

The hypothesis of normality and homoscedasticity of log-transformed data was tested to determine significant differences in the viral RNA2 copy number and in the relative values of Mx transcription between groups as well as between samples collected over time within the same group. The parametric one-way ANOVA test followed by the Fisher’s least significant difference (LSD) test was used. Statistical analysis was performed using the XLSTAT software. Significant differences were considered at values of *p* < 0.05.

## Results

### Challenge 1. Influence of SJNNV exposure on a subsequent RGNNV infection

**Figure 1:**
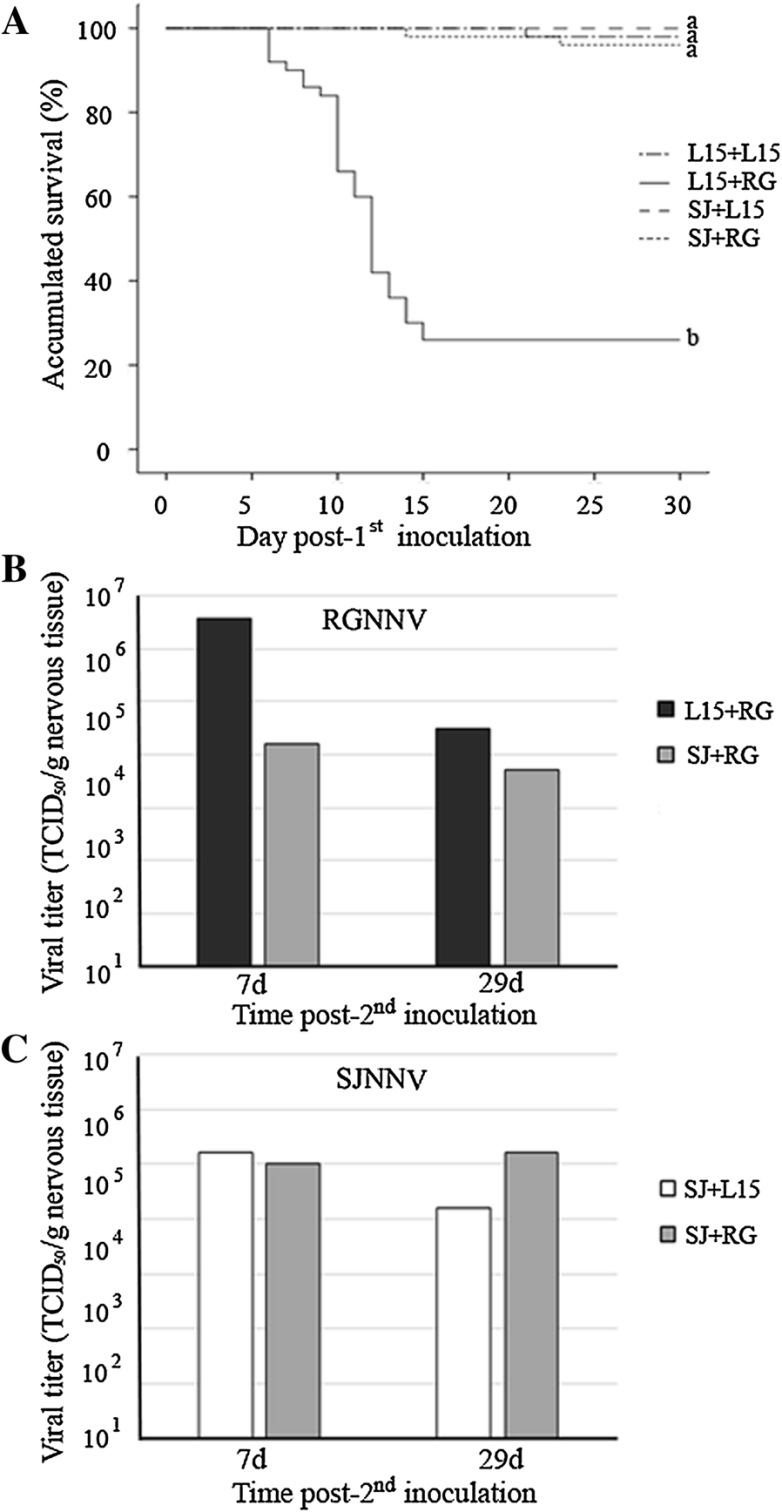
**Kaplan–Meier survival curve, and infective viral particles in fish sampled during challenge 1.** **A** Accumulated survival of European sea bass in different groups within challenge 1. The different letters indicate the significant differences between experimental groups and between challenges 1 and 2 (Figure 4B) (*p* < 0.05). **B** RGNNV titers (TCID_50_/g) in nervous tissue. **C** SJNNV titers (TCID_50_/g) in nervous tissue. Titers in fish from the superinfected group (SJ+RG) were calculated after neutralizing the corresponding genotype.

Single RGNNV inoculation (L15+RG group) resulted in 26% accumulated survival at the end of the experiment (30 days post-first inoculation) (Figure 1A). The first symptoms of the disease appeared at 5 dpi. Mortality onset was at 6 days, and the maximum level of mortality was recorded between 10 and 12 days. In the group previously inoculated with the SJNNV isolate (SJ+RG), the accumulated survival increased up to 96%, since only 2 fish (out of 50) died. No mortality was recorded in the SJNNV-inoculated group (SJ+L15), and only 1 fish died (accidentally) in the negative control group (L15+L15) (Figure 1A).

The typical signs of the disease were only recorded in groups displaying mortalities. In particular, fish in the L15+RG group showed appetite loss, dark pigmentation, abnormal swimming and loss of swim-bladder control, whereas symptoms in the superinfected group (SJ+RG) were less severe such as loss of appetite, dark coloration and slow swimming.

### Viral quantification

Viral titers in fish sampled from the L15+RG group were 3.7 × 10^6^ and 3.2 × 10^4^ TCID_50_/g at 7 and 29 days post-second inoculation, respectively (Figure 1B). The previous SJNNV inoculation (SJ+RG group) resulted in a 10- to100-fold reduction of the RGNNV titers with 1.6 × 10^4^ TCID_50_/g at 7 days and 5 × 10^3^ TCID_50_/g at 29 days post-second inoculation (Figure 1B). In contrast, SJNNV titers in fish from the SJ+L15 group were close to the titers recorded in the superinfected fish (Figure 1C). Thus, in SJNNV-inoculated fish, titers of 1.6 × 10^5^ and 1.6 × 10^4^ TCID_50_/g were recorded at 7 and 29 days post-second inoculation, respectively, whereas titers in the superinfected group were 1 × 10^5^ (at 7 days) and 1.6 × 10^5^ (at 29 days) TCID_50_/g (Figure 1C).

Infective viral particles were also quantified from fish that died at 6 days (the initial stage of the mortality curve) and 12 days post-second inoculation (the exponential phase of the curve) in the L15+RG group. The viral titers in these samples were 2.5 × 10^5^ TCID_50_/g and 1.5 × 10^5^ TCID_50_/g, respectively.

**Figure 2:**
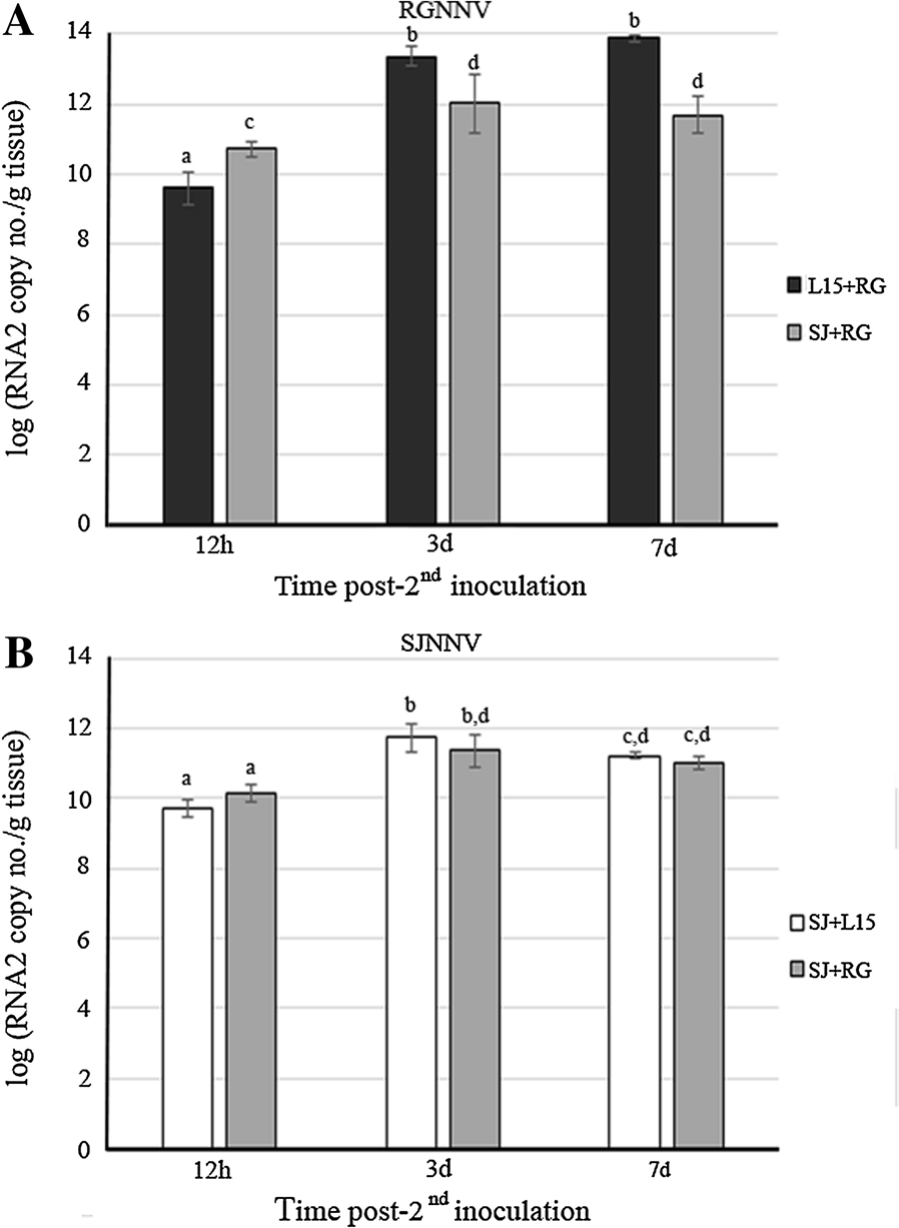
**Viral genome in nervous tissue from fish sampled during challenge 1.** **A** RGNNV RNA2 segment copy number. **B** SJNNV RNA2 segment copy number. Graphics represent the mean values and standard deviation of three samples collected at different time points post-second inoculation from each experimental group. Each sample comprises tissues from three different fish. Different letters indicate the significant differences between groups as well as between samples collected over time within each group (*p* < 0.05).

RGNNV RNA2 copy number in nervous tissue from sampled fish inoculated only with this genotype (L15+RG group) increased significantly (*p* < 0.05) over time, from 9.6 (log RNA2 copy number/g) at 12 h, to 13.3 and 13.9, at 3 and 7 days post-second inoculation, respectively. In the superinfected group (SJ+RG), the log of RGNNV RNA2 copy number also increased over time, from 10.7 at 12 h to 12.0 and 11.7, at 3 and 7 days post-second inoculation, respectively (Figure 2A). However, the previous SJNNV exposure significantly decreased (*p* < 0.05) the RGNNV RNA2 copy number at 3 and 7 days post-second inoculation compared with the values obtained after the single RGNNV inoculation (Figure 2A).

The SJNNV copy number (Figure 2B) in animals from the SJ+L15 group increased significantly (*p* < 0.05) from 12 h (9.7 log RNA2 copy number/g) to 3 days post-second inoculation (11.7 log RNA2 copy number/g). These values were similar (*p* < 0.05) to the values recorded in superinfected fish at all times analyzed (10.1, 11.4 and 11.0 log RNA2 copy number/g, at 12 h, 3 days, and 7 days post-second inoculation, respectively) (Figure 2B).

### Mx transcription quantification

**Figure 3:**
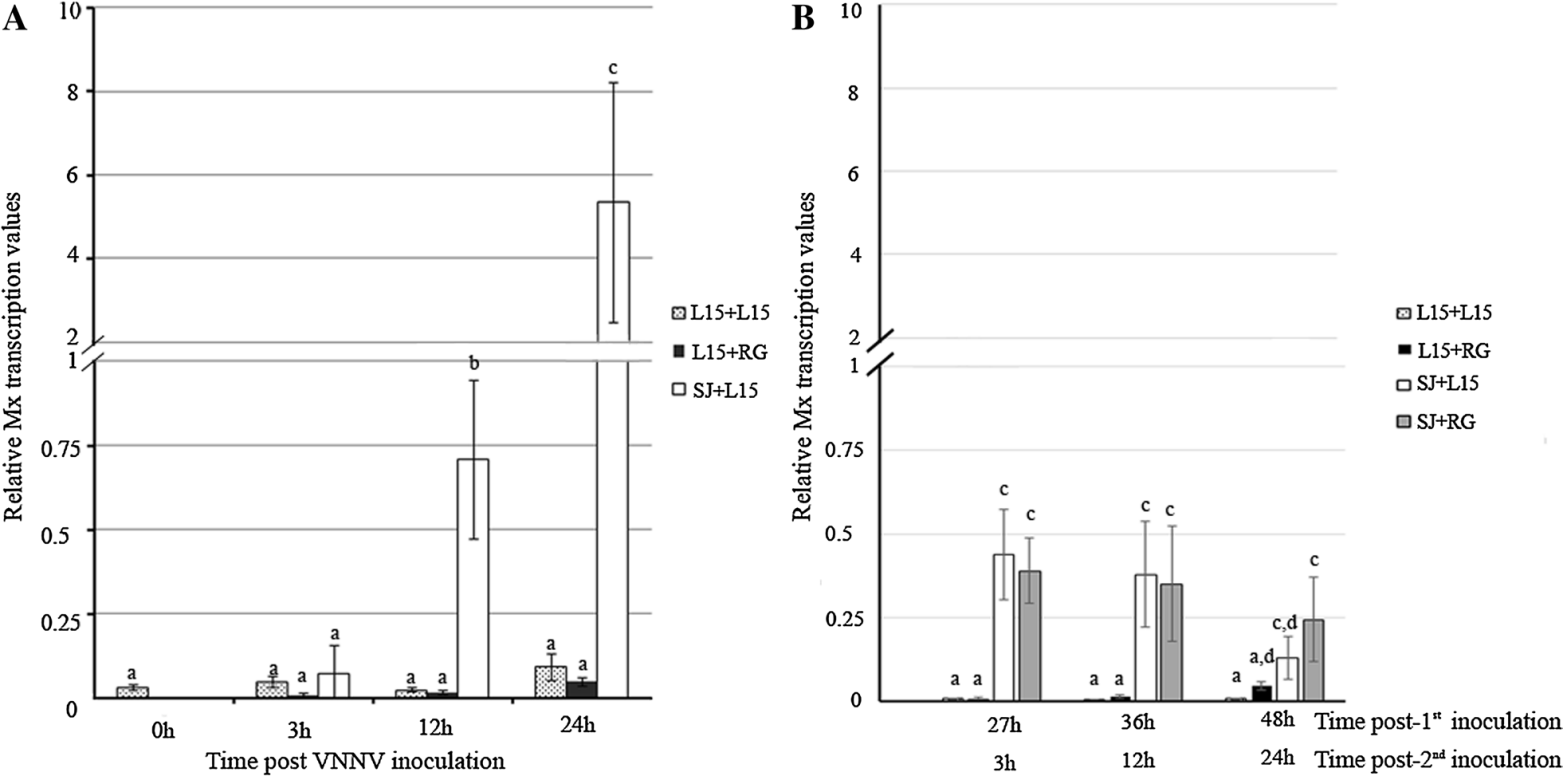
**Relative Mx transcription in head kidney sampled during challenge 1.** **A** Relative Mx mRNA values after single VNNV inoculation. **B** Relative Mx mRNA values after the second inoculation. Graphics represent the mean relative values and standard deviation of three independent samples collected from different experimental groups. Different letters indicate significant differences between groups as well as between samples collected throughout time within each group (*p* < 0.05).

Important differences in the relative values of Mx mRNA were recorded depending on the viral isolate considered. As shown in Figure 3A, the RGNNV isolate (highly pathogenic to sea bass) did not induce Mx transcription in the head kidney at any sampling time considered (L15+RG group), whereas Mx gene transcription was significantly (*p* < 0.05) up-regulated following SJNNV inoculation (SJ+L15 group) at 12 and 24 h post-viral inoculation (Figure 3A). The maximum relative Mx transcription level was at 24 h post-viral inoculation (5.3), which is the time at which the RGNNV inoculation was performed in the superinfected group (SJ+RG) (Figure 3B).

The comparison between the average relative Mx transcription values in SJNNV-inoculated fish (SJ+L15 group: 0.44, 0.38 and 0.13, at 3, 12 and 24 h post-second inoculation, respectively) and superinfected fish (SJ+RG group: 0.39, 0.35 and 0.24, at 3, 12 and 24 h post-second inoculation, respectively) showed that the coexistence of both isolates did not modify the Mx transcription level induced by SJNNV (Figure 3B).

### Challenge 2. Effect of the poly I:C-stimulated IFN I system on RGNNV superinfection

**Figure 4:**
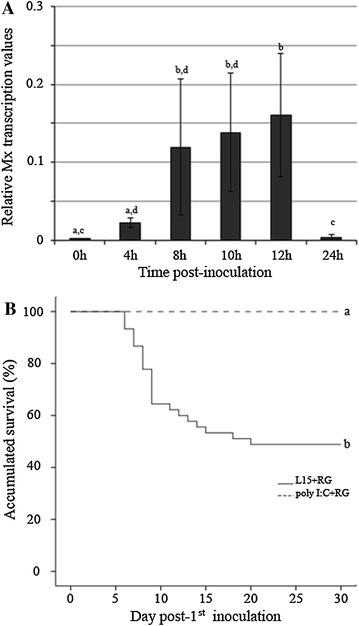
**Kinetics of Mx transcription in head kidney after poly I:C inoculation, and Kaplan-Meier survival curve during challenge 2.** **A** Relative Mx transcription in the head kidney at different times after poly I:C inoculation. The graphics represent the mean relative values of Mx mRNA and standard deviation of three individual samples. Different letters indicate significant differences (*p* < 0.05). **B** Accumulated survival of European sea bass in different groups within challenge 2. Different letters indicate the significant differences between the experimental groups and between challenge 1 (Figure 1a) and challenge 2 (*p* < 0.05).

To confirm that the previous stimulation of the IFN I system protects sea bass against RGNNV infection, juvenile specimens were treated with poly I:C and subsequently challenged with the RGNNV isolate (poly I:C+RG group). The time for the RGNNV inoculation was chosen (at 12 h after poly I:C inoculation) based on the results obtained in a previous challenge in which animals were injected with poly I:C (Figure 4A). The Mx gene relative transcription value at the time of the viral inoculation (12 h post-first inoculation) was 0.45.

The accumulated survival rate of these fish was compared with the rates recorded in the control group (L15+RG) (Figure 4B). The fish in the control group displayed clinical signs of disease beginning at the sixth day, and the highest mortalities were recorded at days 8 and 9 and decreased progressively until day 15. The accumulated survival rate in this group was 48.9%. According to the Breslow test, the accumulated survival rates in challenges 1 (Figure 1A) and 2 (Figure 4B) were not significantly different, with values of *p* < 0.05. No clinical signs or mortalities were recorded in poly I:C-stimulated animals (100% accumulated survival rate) (Figure 4B).

### Challenge 3. Effect of the RGNNV infection on the Mx transcription promoted by poly I:C

The absence of Mx transcription after RGNNV inoculation recorded in challenge 1 (Figure 3A) suggests that this isolate may interfere with the IFN I system. To test this hypothesis, European sea bass were consecutively inoculated with poly I:C and RGNNV (poly I:C+RG group), and the relative Mx transcription values in these fish were compared with the values recorded in fish from the poly I:C+L15 group.

**Figure 5:**
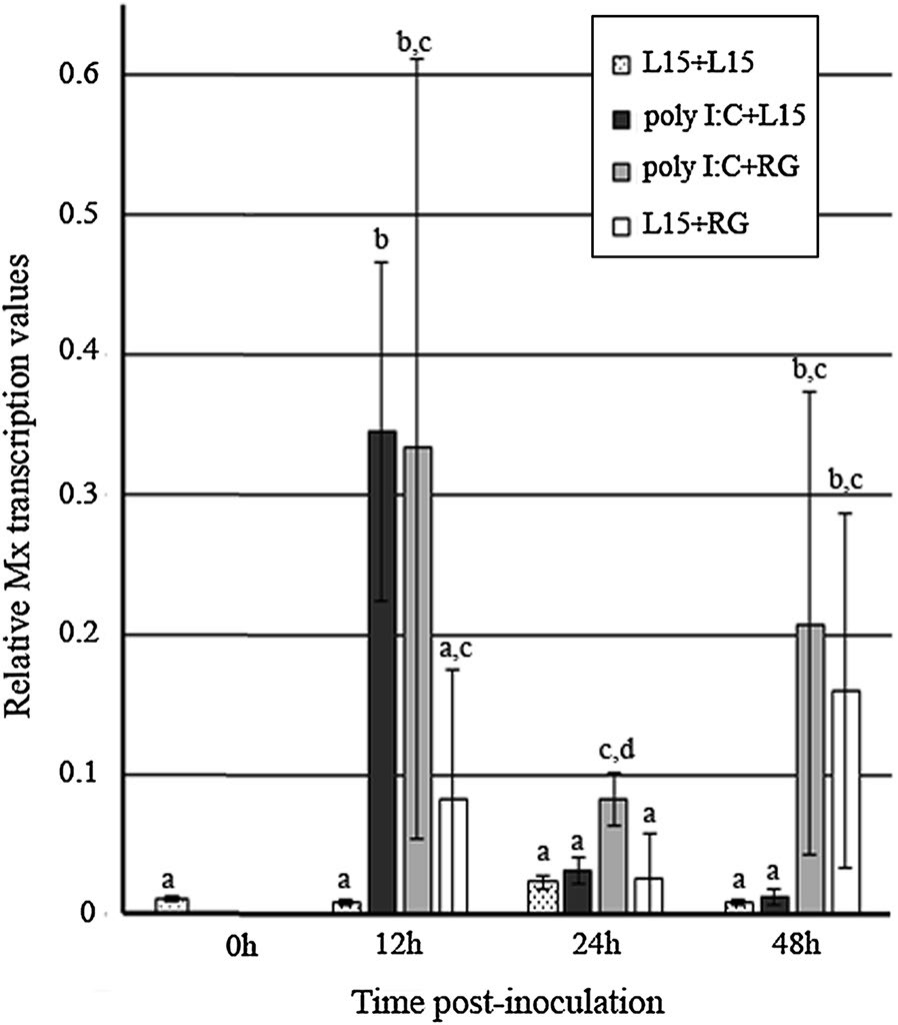
**Relative Mx transcription in head kidney sampled during challenge 3.** The graphics represent the mean Mx mRNA relative values and standard deviation of three individual samples collected at different times post-inoculation. The letters indicate the significant differences between groups as well as between samples collected throughout time within each group (*p* < 0.05).

The results drawn in Figure 5 show similar average relative values of Mx mRNA (*p* < 0.05) in fish from the poly I:C+RG (0.33) and poly I:C+L15 groups (0.34) at 12 hpi. At 24 hpi, the Mx transcription (0.08 relative value) was only recorded in the poly I:C+RG group. In this last group, a second increase in Mx transcription was observed at 48 hpi (0.21 relative value), reaching a mean relative value similar to the value recorded after single RGNNV inoculation (0.16 relative value), which induced Mx transcription only at this sampling time (Figure 5).

## Discussion

Although the RGNNV and SJNNV genotypes have been detected or isolated from European sea bass, RGNNV appears to be the only genotype that causes mass mortality in this fish species, especially affecting larval and juvenile specimens under culture conditions. In addition, these two genotypes have been detected in coexistence in a high number of asymptomatic wild and cultured sea bass [30], suggesting that the RGNNV-SJNNV coinfection may be a frequent event that could down-regulate RGNNV genotype replication.

The present study evaluates the effect of RGNNV-SJNNV coexistence on the replication of each genotype in experimentally challenged juvenile European sea bass, analyzing the possible role of the IFN I-mediated system triggered by the first SJNNV infection on the development of a subsequent RGNNV infection.

To fulfill this purpose, the course of the infection and the relative Mx transcription level were comparatively studied after single (L15+RG and SJ+L15 groups) and dual (SJ+RG group) VNNV inoculation in challenge 1.

In the L15+RG group, the cumulative mortality was 74%, which was above the range most frequently reported for sea bass (2–50%) depending on the viral isolate, the inoculation method, and the fish weight [8, 26, 31]. The presence of viral infective particles in dead fish in this group was confirmed by the viral titer quantification. The highest viral titer was obtained in fish dying during the exponential phase of the mortality curve, when clinical signs were more evident. This result supported previous findings obtained after IM inoculation [26] or bath infection [32] in European sea bass. Moreover, a high level of viral replication has been demonstrated in randomly sampled fish by qPCR and TCID_50_. Particularly, the highest number of viral genome copies (13.9 log RNA2 copy number/g) and viral titer (3.7 × 10^6^ TCID_50_/g) were obtained at 7 days when the first symptoms and mortalities were recorded. Previously, Lopez-Jimena et al. [26] reported similar viral titers (ca. 1 × 10^6^ TCID_50_/g) at 10 dpi in the nervous tissue of RGNNV-inoculated European sea bass.

In the SJ+L15 group, the accumulated survival rate was 100%, confirming the low pathogenicity of this genotype to European sea bass, as previously reported [8, 33]. In addition, our results regarding the viral multiplication (determined by qPCR and titration) support the findings reported by Souto et al. [33], showing that the SJNNV isolate replicates less efficiently than the RGNNV isolate in European sea bass nervous tissue.

Nodavirus multiplication in animals showing no signs of disease or mortality has been previously described in several fish species [27, 34–38]. In particular, in gilthead sea bream, RGNNV shows low replication levels and induces high Mx transcription levels in brain and/or head kidney [27], which has been considered a main factor involved in the resistance of this fish species to nodavirus infections.

In the SJ+RG group, the accumulated survival rate was 96%, and the quantification of viral RNA shows that the coexistence of both isolates did not affect SJNNV replication, whereas RGNNV replication was clearly decreased at 3 and 7 days post-second inoculation.

Therefore, these results suggest the induction of an antiviral state after the injection of an SJNNV isolate, which protects juvenile European sea bass against RGNNV infection and compromises RGNNV replication in nervous tissue. Similar results have been reported in rainbow trout (*Oncorhynchus mykiss*) inoculated with the infectious hematopoietic necrosis virus (IHNV) and the infectious pancreatic necrosis virus (IPNV) during coinfection [18] and superinfection [12] as well as in IPNV-infected Atlantic salmon (*Salmo salar*) superinfected with the salmon anemia virus (SAV) [11]. Furthermore, Pakingking et al. [19] demonstrated the negative effect of a non-lethal aquabirnavirus (ABV) on RGNNV replication in seven-band groupers (*Epinephelus septemfasciatus*).

Although the results obtained in this study clearly show the negative effect of SJNNV exposure on RGNNV replication and on the course of its infection in juvenile European sea bass, these findings were not in concordance with the results recorded by Lopez-Jimena et al. [21] using an in vitro approach. According to these authors, RGNNV replication on E-11 cells is favored by the presence of SJNNV, whereas there is a negative effect of RGNNV on SJNNV replication. However, the possible cause of this viral interference was not considered, and the discrepancies in the results obtained emphasize the importance of analyzing the differences between both isolates regarding the interaction with each specific host, in which the innate immune system plays an important role.

The negative effect on RGNNV replication shown in superinfected sea bass may be due to the stimulation of the host IFN I-mediated immune system or other antiviral factor by the first-inoculated virus [11, 12]. For this reason, we compared the IFN I system response triggered by both VNNV isolates after single inoculation and superinfection (challenge 1). Mx gene transcription has been evaluated as a reporter of IFN I system stimulation. The findings of this study revealed important differences in the host-virus interaction depending on the nodavirus isolate considered. Thus, the SJNNV isolate, which replicates less efficiently than the RGNNV isolate in this fish species, is a potent inducer of sea bass Mx transcription, even stronger than poly I:C. In contrast, RGNNV single inoculation did not result in Mx gene transcription between 3 and 24 h post-viral inoculation. In addition, the second RGNNV inoculation (SJ+RG group) did not alter the Mx gene transcription pattern induced by the SJNNV isolate at any time tested.

Therefore, a low level of SJNNV-isolate RNA induces an early and strong IFN I response, producing an anti-viral state, which may further prevent viral replication and may be a factor in determining the low pathogenicity of the SJNNV isolates to European sea bass. Furthermore, this IFN I-system induction may have been responsible for the down-regulation of RGNNV replication recorded in the superinfected group.

To our knowledge, this work is the first report of Mx transcription induced by SJNNV in head kidney from European sea bass, whereas previous reports have described Mx transcription in this fish species following RGNNV infection. Scapigliati et al. [28] and Novel et al. [39] recorded early Mx transcription (6 hpi); however, Chaves-Pozo et al. [27] did not detect Mx mRNA before 24 hpi. Differences in fish age, viral isolates, and methodology may explain these discrepancies.

The relationship between Mx transcription and the generation of an antiviral state has been previously suggested for Japanese flounder (*Paralichthys olivaceus*) [40] and seven-band groupers [19]. Moreover, Chen et al. [13] demonstrated that the over-expression of Mx in the seven-band grouper results in reduced viral yields, playing a key role in cellular resistance to nodavirus infection. In contrast, Wu et al. [14] suggested that VNNV RNA synthesis is reduced by the Mx-RdRp interaction in barramundi (*Lates calcarifer*).

The prevention of RGNNV infection reported in challenge 1 may not be directly related to the IFN I system stimulation triggered by SJNNV but may instead be related to the SJNNV-RGNNV competition for the target nervous cells in superinfected fish. To confirm the role of the IFN I system against RGNNV infection in European sea bass, a second challenge was performed in which the primary SJNNV inoculation was replaced by the injection of a synthetic IFN I system inducer (poly I:C+RG group) (challenge 2).

The accumulated survival rate in animals inoculated only with the RGNNV isolate (L15+RG group) was 48.9%, which, according to the Breslow test, was not significantly different from the findings recorded in challenge 1, despite the different weight of the animals used in each challenge. Previous poly I:C stimulation (poly I:C+RG group) resulted in a drastic increase of the accumulated survival rate (100%), indicating that the IFN I system stimulated by poly I:C elicits an anti-RGNNV state in European sea bass.

The protective effect promoted by poly I:C has been previously reported in several fish species against different viruses [16, 17]. In particular, the anti-RGNNV state has been demonstrated in the poly I:C-treated seven-band grouper [41]. In a recent study, Thanasaksiri et al. [42] demonstrated that poly I:C stimulation reduces RGNNV replication in the seven-band grouper, which supports the results obtained in challenge 1 after SJNNV inoculation. Based on these results, the IFN I system, induced by poly I:C or SJNNV injection, is thought to play an important role in protecting European sea bass against RGNNV infection.

The absence of Mx transcription after RGNNV inoculation recorded in challenge 1 suggests that the RGNNV isolate displays antagonistic mechanisms against the IFN I system. Furthermore, Mx transcription triggered by SJNNV is not subsequently altered by RGNNV superinfection. However, RGNNV replication could affect the simultaneous stimulation of IFN I. To verify this possible effect, the animals were consecutively inoculated with poly I:C and RGNNV (poly I:C+RG group), and the relative Mx transcription values in these fish were compared with the values recorded in fish from the poly I:C+L15 group (challenge 3).

According to the results obtained in challenge 3, RGNNV multiplication did not reduce Mx transcription induced by poly I:C at any tested time; however, the negative interference of RGNNV with other ISG to evade the innate host defense cannot be ruled out. This finding corroborated the result obtained in challenge 1 (SJ+RG group), which shows that RGNNV does not interfere with the Mx transcription triggered by the previous SJNNV infection. Although antagonistic mechanisms that interfere with the IFN I response have been described for other fish viruses [43–47], they have not been reported in any VNNV isolate to date.

Interestingly, the Mx gene was transcribed 48 h after RGNNV inoculation (L15+RG group), although at a low level compared with SJNNV-triggered induction. This finding indicates that this isolate induces Mx transcription later than poly I:C and SJNNV, suggesting that a high level of RGNNV multiplication may be required for this isolate to induce the IFN I system response in sea bass. In fact, at 24 hpi, a higher level of Mx transcription was observed in the poly I:C+RG group compared with the transcription recorded after the single poly I:C inoculation. This induction most likely is due to a synergic effect between the remaining poly I:C and the beginning of RGNNV replication. Moreover, the absence of poly I:C and the higher virus load at 48 hpi could explain the similar Mx transcription recorded at this sampling time in fish from the poly I:C+L15 and poly I:C+RG groups. In a previous study, Nishizawa et al. [41] demonstrated that the poly I:C injection in seven-band grouper at 2 and 4 days after RGNNV inoculation does not have curative effects, which, according to these authors, may be due to the high virus load. Similarly, in our study the high RGNNV load at 48 hpi, when the Mx transcription was induced after RGNNV infection, may have made the IFN I system response ineffective against the viral infection.

In summary, this work is a comprehensive study in which the role of the IFN I system in controlling VNNV infections in European sea bass is demonstrated using different in vivo approaches. This study demonstrates Mx transcription stimulation in head kidney following SJNNV inoculation, and the induction of an anti-RGNNV state following the injection of SJNNV and poly I:C. In addition, the RGNNV isolate does not negatively interfere with Mx transcription in the European sea bass and induces the IFN I-mediated system later that poly I:C and SJNNV, which may be related to the high pathogenicity of this genotype in this fish species.

## Abbreviations

ABV: Aquabirnavirus
BFNNV: Barfin flounder nervous necrosis virus
CP: Capsid protein
CPE: Cytopathic effect
FBS: Fetal bovine serum
IFN I: Type I interferon
IHNV: Infectious hematopoietic necrosis virus
IM: Intramuscular
IPNV: Infectious pancreatic necrosis virus
ISG: Interferon stimulated gene
L15: Leibovitz medium
Pi: Post-inoculation
Poly I:C: Polyinosinic-polycystidylic acid
RdRp: RNA-dependent RNA-polymerase
RGNNV: Red-spotted grouper nervous necrosis virus
SAV: Salmon anemia virus
SJNNV: Striped jack nervous necrosis virus
TCID_50_: 50% tissue culture infectious dose
TPNNV: Tiger puffer nervous necrosis virus
VNN: Viral nervous necrosis
VNNV: Viral nervous necrosis virus

## References

[1] Nakai T, Mori K, Sugaya T, Nishioka T, Mushiake K, Yamashita H (2009). Current knowledge on viral nervous necrosis (VNN) and its causative betanodaviruses. Isr Aquacult Bamid.

[2] Mori K, Nakai T, Muroga K, Arimoto M, Mushiake K, Furusawa I (1992). Properties of a new virus belonging to *nodaviridae* found in larval striped jack (*Pseudocaranx dentex*) with nervous necrosis. Virology.

[3] Nishizawa T, Furuhashi M, Nagai T, Nakai T, Muroga K (1997). Genomic classification of fish nodaviruses by molecular phylogenetic analysis of the coat protein gene. Appl Environ Microbiol.

[4] Thiery R, Cozien J, De Boisseson C, Kerbart-Boscher S, Nevarez L (2004). Genomic classification of new betanodavirus isolates by phylogenetic analysis of the coat protein gene suggests a low host-fish species specificity. J Gen Virol.

[5] Cutrin JM, Dopazo CP, Thiery R, Leao P, Olveira JG, Barja JL, Bandin I (2007). Emergence of pathogenic betanodaviruses belonging to the SJNNV genogroup in farmed fish species from the Iberian Peninsula. J Fish Dis.

[6] Olveira JG, Soares F, Engrola S, Dopazo CP, Bandin I (2008). Antemortem versus postmortem methods for detection of betanodavirus in Senegalese sole (*Solea senegalensis*). J Vet Diagn Invest.

[7] Olveira JG, Souto S, Dopazo CP, Thiery R, Barja JL (2009). Comparative analysis of both genomic segments of betanodaviruses isolated from epizootic outbreaks in farmed fish species provides evidence for genetic reassortment. J Gen Virol.

[8] Vendramin N, Toffan A, Mancin M, Cappellozza E, Panzarin V, Bovo G, Cattoli G, Capua I, Terregino C (2014). Comparative pathogenicity study of ten different betanodavirus strains in experimentally infected European sea bass, *Dicentrarchus labrax* (L.). J Fish Dis.

[9] Lopez-Jimena B, Cherif N, Garcia-Rosado E, Infante C, Cano I, Castro D, Hammami S, Borrego JJ, Alonso MC (2010). A combined RT-PCR and dot-blot hybridization method reveals the coexistence of SJNNV and RGNNV betanodavirus genotypes in wild meagre (*Argyrosomus regius*). J Appl Microbiol.

[10] Toffolo V, Negrisolo E, Maltese C, Bovo G, Balvedere P, Colombo L, Dalla Valle L (2007). Phylogeny of betanodaviruses and molecular evolution of their RNA polymerase and coat proteins. Mol Phylogenet Evol.

[11] Johansen LH, Sommer AI (2001). Infectious pancreatic necrosis virus infection in Atlantic salmon *Salmo salar* post-smolts affects the outcome of secondary infections with infectious salmon anaemia virus or *Vibrio salmonicida*. Dis Aquat Organ.

[12] Byrne N, Castric J, Lamour F, Cabon J, Quentel C (2008). Study of the viral interference between infectious pancreatic necrosis virus (IPNV) and infectious haematopoietic necrosis virus (IHNV) in rainbow trout (*Oncorhynchus mykiss*). Fish Shellfish Immunol.

[13] Chen YM, Su YL, Shie PS, Huang SL, Yang HL, Che TY (2008). Grouper Mx confers resistance to nodavirus and interacts with coat protein. Dev Comp Immunol.

[14] Wu YC, Lu YF, Chi SC (2010). Anti-viral mechanism of barramundi Mx against betanodavirus involves the inhibition of viral RNA synthesis through the interference of RdRp. Fish Shellfish Immunol.

[15] Kochs G, Reichelt M, Danino D, Hinshaw JE, Haller O (2005). Assay and functional analysis of dynamin-like Mx proteins. Methods Enzymol.

[16] Robertsen B (2006). The interferon system of teleost fish. Fish Shellfish Immunol.

[17] Robertsen B (2008). Expression of interferon and interferon-induced genes in salmonids in response to virus infection, interferon-inducing compounds and vaccination. Fish Shellfish Immunol.

[18] Alonso M, Rodriguez Saint-Jean S, Perez-Prieto SI (2003). Virulence of infectious hematopoietic necrosis virus and infectious pancreatic necrosis virus coinfection in rainbow trout (*Oncorhynchus mykiss*) and nucleotide sequence analysis of the IHNV glycoprotein gene. Arch Virol.

[19] Pakingking R, Mori K, Sugaya T, Oka M, Okinaka Y, Nakai T (2005). Aquabirnavirus-induced protection of marine fish against piscine nodavirus infection. Fish Pathol.

[20] Kim HJ, Oseko N, Nishizawa T, Yoshimizu M (2009). Protection of rainbow trout from infectious hematopoietic necrosis (IHN) by injection of infectious pancreatic necrosis virus (IPNV) or poly(I:C). Dis Aquat Organ.

[21] Lopez-Jimena B, Garcia-Rosado E, Infante C, Castro D, Borrego JJ, Alonso MC (2014). Effect of the coexistence on the replication of striped jack nervous necrosis virus (SJNNV) and red-spotted grouper nervous necrosis virus (RGNNV) using an in vitro approach. J Appl Ichthyol.

[22] Iwamoto T, Nakai T, Mori K, Arimoto M, Furusawa I (2000). Cloning of the fish cell line SSN-1 for piscine nodaviruses. Dis Aquat Organ.

[23] Reed LJ, Muench H (1938). A simple method of estimating 50 per cent end-points. Am J Hyg.

[24] RD 53/2013, BOE no. 34. Normas básicas aplicables para la protección de los animales utilizados en experimentación y otros fines científicos, incluyendo la docencia. Accessed 23 Mar 2015

[25] Van Belle G, Fisher LD, Heagerty PJ, Lumley T (2004). Biostatistics: a methodology for the health sciences.

[26] Lopez-Jimena B, Alonso MC, Thompson KD, Adams A, Infante C, Borrego JJ, Garcia-Rosado E (2011). Tissue distribution of Red spotted grouper nervous necrosis virus (RGNNV) genome in experimentally infected juvenile European seabass (*Dicentrarchus labrax*). Vet Microbiol.

[27] Chaves-Pozo E, Guardiola FA, Meseguer J, Esteban MA, Cuesta A (2012). Nodavirus infection induces a great innate cell-mediated cytotoxic activity in resistant, gilthead seabream, and susceptible, European sea bass, teleost fish. Fish Shellfish Immunol.

[28] Scapigliati G, Buonocore F, Randelli E, Casani D, Meloni S, Zarletti G, Tiberi M, Pietretti D, Boschi I, Manchado M, Martin-Antonio B, Jimenez-Cantizano R, Bovo G, Borghesan F, Lorenzen N, Einer-Jensen K, Adams S, Thompson K, Alonso C, Bejar J, Cano I, Borrego JJ, Alvarez MC (2010). Cellular and molecular immune responses of the sea bass (*Dicentrarchus labrax*) experimentally infected with betanodavirus. Fish Shellfish Immunol.

[29] Livak KJ, Schmittgen TD (2001). Analysis of relative gene expression data using real-time quantitative PCR and the 2^−ΔΔCt^ method. Methods.

[30] Lopez-Jimena B (2012) Pathogenesis studies and detection of betanodavirus in marine fish species from the Iberian Peninsula. PhD thesis. Universidad de Málaga

[31] Skliris GP, Richards RH (1999). Induction of nodavirus disease in seabass, *Dicentrarchus labrax*, using different infection models. Virus Res.

[32] Dalla Valle L, Toffolo V, Lamprecht M, Maltese C, Bovo G, Belvedere P, Colombo L (2005). Development of a sensitive and quantitative diagnostic assay for fish nervous necrosis virus based on two-target real-time PCR. Vet Microbiol.

[33] Souto S, Lopez-Jimena B, Alonso MC, Garcia-Rosado E, Bandin I (2015). Experimental susceptibility of European sea bass and Senegalese sole to different betanodavirus isolates. Vet Microbiol.

[34] Castric J, Thiery R, Jeffroy J, de Kinkelin P, Raymond JC (2001). Sea bream *Sparus aurata*, an asymptomatic contagious fish host for nodavirus. Dis Aquat Organ.

[35] Grove S, Johansen R, Dannevig BH, Reitan LJ, Ranheim T (2003). Experimental infection of Atlantic halibut *Hippoglossus hippoglossus* with nodavirus: tissue distribution and immune response. Dis Aquat Organ.

[36] Cherif N, Thiery R, Castric J, Biacchesi S, Bremont M, Thabti F, Limem L, Hammami S (2009). Viral encephalopathy and retinopathy of *Dicentrarchus labrax* and *Sparus aurata* farmed in Tunisia. Vet Res Commun.

[37] Korsnes K, Karlsbakk E, Devold M, Nerland AH, Nylund A (2009). Tissue tropism of nervous necrosis virus (NNV) in Atlantic cod, *Gadus morhua* L., after intraperitoneal challenge with a virus isolate from diseased Atlantic halibut, *Hippoglossus hippoglossus* (L.). J Fish Dis.

[38] Lopez-Muñoz A, Sepulcre MP, Garcia-Moreno D, Fuentes I, Bejar J, Manchado M, Alvarez MC, Meseguer J, Mulero V (2012). Viral nervous necrosis virus persistently replicates in the central nervous system of asymptomatic gilthead seabream and promotes a transient inflammatory response followed by the infiltration of IgM+ B lymphocytes. Dev Comp Immunol.

[39] Novel P, Fernandez-Trujillo MA, Gallardo-Galvez JB, Cano I, Manchado M, Buonocore F, Randelli E, Scapigliati G, Alvarez MC, Bejar J (2013). Two Mx genes identified in European sea bass (*Dicentrarchus labrax*) respond differently to VNNV infection. Vet Immunol Immunopathol.

[40] Pakingking R, Takano R, Nishizawa T, Mori KI, Lida Y, Arimoto M, Muroga K (2003). Experimental coinfection with aquabirnavirus and viral hemorrhagic septicemia virus (VHSV), *Edwardsiella tarda* or *Streptococcus iniae* in Japanese flounder (*Paralichthys olivaceus*). Fish Pathol.

[41] Nishizawa T, Takami I, Kokawa Y, Yoshimizu M (2009). Fish immunization using a synthetic double-stranded RNA Poly (I:C), an interferon inducer, offers protection against RGNNV, a fish nodavirus. Dis Aquat Organ.

[42] Thanasaksiri K, Sakai N, Yamashita H, Hirono I, Kondo H (2014). Influence of temperature on Mx gene expression profiles and the protection of sevenband grouper, *Epinephelus septemfasciatus*, against red-spotted grouper nervous necrosis virus (RGNNV) infection after poly (I:C) injection. Fish Shellfish Immunol.

[43] McBeath AJ, Snow M, Secombes CJ, Ellis AE, Collet B (2007). Expression kinetics of interferon and interferon-induced genes in Atlantic salmon (*Salmo salar*) following infection with infectious pancreatic necrosis virus and infectious salmon anaemia virus. Fish Shellfish Immunol.

[44] Garcia-Rosado E, Markussen T, Kileng O, Baekkevold ES, Robertsen B, Mjaaland S, Rimstad E (2008). Molecular and functional characterization of two infectious salmon anaemia virus (ISAV) proteins with type I interferon antagonizing activity. Virus Res.

[45] Skjesol A, Aamo T, Hegseth MN, Robertsen B, Jørgensen JB (2009). The interplay between infectious pancreatic necrosis virus (IPNV) and the IFN system: IFN signaling is inhibited by IPNV infection. Virus Res.

[46] Kim MS, Kim KH (2012). Effects of NV gene knock-out recombinant viral hemorrhagic septicemia virus (VHSV) on Mx gene expression in epithelioma papulosum cyprini (EPC) cells and olive flounder (*Paralichthys olivaceus*). Fish Shellfish Immunol.

[47] Kim MS, Kim KH (2013). The role of viral hemorrhagic septicemia virus (VHSV) NV gene in TNF-α- and VHSV infection-mediated NF-κB activation. Fish Shellfish Immunol.

